# Beyond Crisis: Understandings of Vulnerability and Its Consequences in Relation to Intimate Partner Violence

**DOI:** 10.1007/s12142-023-00687-3

**Published:** 2023-06-06

**Authors:** Nesa Zimmermann

**Affiliations:** University of Neuchâtel, Avenue du Premier-Mars 26, 2000 Neuchâtel, Switzerland

**Keywords:** Domestic violence, Intimate partner violence, COVID-19, Pandemic, Vulnerability, European Convention on Human Rights, European Court of Human Rights, Istanbul Convention, Positive obligations, Due diligence obligations, Crisis discourse

## Abstract

This article takes a closer look at intimate partner violence (IPV) and its semantical, political, and legal interactions with crisis and crisis discourse. Starting from the fact that IPV has been called a “shadow pandemic” and a “hidden crisis”, the article conceptualizes two parallel phenomena: how the COVID-19 pandemic — and crises in general — impact on IPV by exacerbating vulnerabilities and how crisis discourse has been mobilized to argue for a responsive state and strong positive obligations to combat and reduce IPV. The article then draws a parallel between crisis discourse and vulnerability reasoning, analyzing how vulnerability has played a similar role within the case law of the European Court of Human Rights (ECtHR) and led the latter to develop a consistent strand of case law concretizing states’ positive obligations. The article also takes a critical stance, examining the risks of crisis discourse and vulnerability when viewed through a crisis lens. To counter these risks, it argues for a nuanced, structural, and dynamic understanding of vulnerability and a focus on resilience-building institutions and mechanisms. Within the ECtHR case law, this signifies elaborating upon the already existing positive obligations, including by taking inspiration from the Council of Europe Convention on preventing and combating violence against women and domestic violence (Istanbul Convention). Such an approach is necessary to leave behind the emergency time usually associated with crises and work toward lasting structural change.

## Introduction

In the wake of the global COVID-19 crisis, intimate partner violence (IPV) has been termed a “pandemic within the pandemic” (Evans et al. 2020, p. 2302), a “shadow pandemic” (UN Women 2021a), a “hidden pandemic” (Xue et al. 2020) or a “hidden crisis” (End Violence against Children 2020).^1^ These expressions operate on two levels.

First, they highlight the ways in which the COVID-19 pandemic has impacted IPV. Indeed, emerging data indicates that, in many countries around the world, IPV has soared during the pandemic (UN Women 2021a, p. 5). Thus, while reminding us of our vulnerability as a “universal, inevitable, enduring aspect of the human condition” (Fineman 2008, p. 8), the pandemic has highlighted that vulnerabilities are unequally distributed.^2^ While vulnerabilities in relation to IPV are due to multiple factors, they are exacerbated by state responses aimed at tackling the “visible” COVID-19 crisis, such as lockdowns, closure of public services, or limited access to non-vital health care services. The health crisis induced by COVID-19 thus illustrates how different vulnerabilities interact and exacerbate each other and how state responses to some vulnerabilities can create others. It also raises the question of state obligations — that is, to what extent these exacerbated vulnerabilities call for heightened state responsibilities in the form of due diligence or positive obligations.^3^

Second, beyond COVID-19, expressions such as “shadow pandemic” are used to underline that IPV is a public health issue (hence, a pandemic), albeit one that still receives too little attention, thus remaining in the shadows of public policy.^4^ The term “crisis” serves a similar purpose, additionally highlighting the urgency of the issue (Abrams 2018, p. 751). Hence, crisis framing both visibilizes IPV and argues for the deployment of state resources to combat it. Similarly, recognizing someone’s particular vulnerability can be used to argue for a responsive state, for example in the form of strengthened positive obligations. However, while being powerful rhetorical tools, crisis and vulnerability framings are not without drawbacks. Specifically, crisis discourse risks reinforcing stereotypical views of victimization and might lead to privileging repressive state action over more holistic, social, and preventive approaches to IPV. By doing so, the crisis frame may create additional, pathogenic vulnerabilities.^5^ Vulnerability reasoning carries similar risks. However, I argue that these risks exist precisely because, and when, vulnerability is narrowly understood through a crisis lens, labeling some groups and persons as vulnerable to argue for their protection. In contrast, a relational and dynamic understanding of vulnerability, which focuses on the underlying structural causes, arguably has a more long-term effect, addressing the root causes of human rights violations by creating resilience-building institutions.

This article examines the way crisis and vulnerability narratives have been mobilized to call for more robust state action and strengthen states’ obligations in addressing IPV, drawing on theoretical concepts as well as human rights practice. For the latter, I focus on the case law of the European Court of Human Rights (ECtHR), which serves well to illustrate the links between vulnerability reasoning and positive obligations. The article is structured as follows. “IPV: a human rights issue” defines IPV and the way it is captured by international and regional human rights instruments. “IPV in times of crisis: exacerbating vulnerabilities” argues that the pandemic — and measures aimed at containing it — have exacerbated preexisting vulnerabilities, aggravating IPV. It also draws on scholarship linking crisis and vulnerabilities. “Crisis framing: exposing vulnerabilities” shows how a sustained and concerted policy-making effort relying on crisis discourse has contributed to exposing the issue of IPV and highlighting state responsibility to combat it, linking the phenomenon within the wider context of crisis narrative. “Vulnerability rhetoric and positive obligations” dives deeper into state responsibilities to combat IPV. Taking the ECtHR as an example, it illustrates the role of vulnerability reasoning in defining states’ positive obligations. “Vulnerability seen through a crisis lens” draws a parallel between vulnerability rhetoric as present in ECtHR case law and crisis narratives and critically examines the pitfalls and risks of crisis discourse. “Beyond crisis discourse: a nuanced approach to structural vulnerabilities” suggests ways to overcome these drawbacks by developing a more nuanced understanding of vulnerabilities.

## IPV: A Human Rights Issue

IPV can be defined as any act of violence committed by a current or former partner or spouse (European Institute for Gender Equality [EIGE] 2017, p. 7; EIGE 2021, p. 4). Importantly, IPV is not limited to physical violence, but more generally encompasses “all acts of physical, sexual, psychological, or economic violence […] between former or current spouses or partners, regardless of whether the perpetrator shares or has shared the same home as the victim” (Article 3 let. b Istanbul Convention; T.M. et C.M. v. Moldova, para. 47; T.M. et C.M. v. Moldova, para. 47; Volodina v. Russia, para. 60; Buturugă v. Romania, paras 38 and 67; EIGE 2017, p. 7; EIGE 2021, p. 4).^6^ Two aspects are worth highlighting. First, IPV is not limited to violence committed by current partners, but also comprehends violence perpetrated by ex-partners or during the separation process, which happens all too frequently (Meyersfeld 2012, pp. 128–129). Second, the definition’s material scope is explicitly not limited to acts of sexual and physical violence, but also encompasses psychological and economic violence, including cyber violence (Buturugă v. Romania, paras 40–42 and 74; Volodina v. Russia No. *2*, paras 48–49; Zimmermann 2022, p. 507). This is crucial: previous scholarly literature has forcefully criticized responses to domestic violence that disproportionately focused on physical violence (Meyersfeld 2004, pp. 387–389; Schneider 2000, p. 12). The definition also captures a grim reality: within the European Union, it is estimated that on top of the 20% of women^7^ experiencing sexual or physical violence from their partner at some point, a staggering 43% experience psychological violence, including threats and controlling behavior (FRA 2014, pp. 29 and 71).

Whereas IPV concerns persons of all genders and sexual orientations, including persons in same-sex relationships, it predominantly affects women. Statistics by the World Health Organization (WHO) indicate that one third of all women worldwide have experienced IPV (WHO 2021, p. 1). Accordingly, many specialized instruments, such as the Inter-American Convention on the Prevention, Punishment, and Eradication of Violence against Women (Belém do Pará Convention, articles 1 and 2) and the Protocol to the African Charter on Human and Peoples’ Rights on the Rights of Women in Africa (Maputo Protocol, article 1 lit. j.) address IPV within the wider phenomenon of gender-based violence (Sjöholm 2018, p. 390; Sosa 2017, pp. 11–12).^8^ In contrast, the Council of Europe Convention on preventing and combating violence against women and domestic violence (Istanbul Convention, articles 2 and 3) deals with IPV separately from gender-based violence, and the ECtHR has repeatedly stressed that men and boys can also be victims (Opuz v. Turkey, para. 123; Civek v. Turkey, para. 50; see also Council of Europe 2011, para. 27). However, the Istanbul Convention explicitly recognizes that domestic violence, including IPV, is a form of violence against women that not only disproportionately affects women, but is also deeply rooted in patriarchal power structures (Article 2, paras 1 and 2; Council of Europe 2011, paras 19 and 44). After some initial reluctance, the ECtHR has similarly recognized the phenomenon’s gendered nature (Volodina v. Russia, para. 110; Tkhelidze v. Georgia, paras 76–77; Sjöholm 2018, p. 403). This has notably allowed the ECtHR to find violations of the prohibition of discrimination, shed light on discriminatory attitudes or policies, and strengthen positive obligations related to IPV (Zimmermann 2022, pp. 553–567).^9^

Today, and after a decades-long struggle by feminist scholars and women’s rights groups alike (Charlesworth 1994, pp. 71–73; Bunch 1990, pp. 489–490), IPV is recognized as a major human rights issue both internationally and regionally. On the international level, this recognition has gained momentum with the adoption of General Recommendation No. 19 by the Committee on the Elimination of Discrimination against Women (CEDAW), which explicitly identifies IPV and other forms of gender-based violence as a form of discrimination.^10^ Ever since, important awareness-raising and normative efforts have taken place, notably via the work of several other United Nations (UN) bodies or representatives, such as the UN Special Rapporteur on violence against women and girls,^11^ the Office of the Hight Commissioner on Human Rights (OHCHR) working group on discrimination against women and girls,^12^ as well as a myriad of international and regional coordination and policy efforts.^13^ The adoption of CEDAW General Recommendation No. 35 in 2017 represented another milestone.^14^ On the regional levels, the Belém do Pará Convention, the Maputo Protocol, the Istanbul Convention, as well as the practice of regional human rights bodies define state obligations to combat IPV. Recognizing IPV as a human rights issue and violation of several human rights^15^ necessitated a paradigmatic shift — namely accepting that “the private is political” and that states are, to a certain extent, responsible for human rights violations perpetrated by individuals in the private sphere.^16^ The exact scope of these due diligence and positive obligations continues to be defined and negotiated; as I will show below using ECtHR case law as an example, vulnerability reasoning plays an important role in this development. However, first I will examine two parallel phenomena that have occurred during the COVID-19 pandemic: measures aimed at combating the crisis have exacerbated existing vulnerabilities; at the same time, crisis rhetoric has also drawn attention to these vulnerabilities and called for a more robust response.

## IPV in Times of Crisis: Exacerbating Vulnerabilities

The quantitative impact of the COVID-19 crisis on gender-based violence and IPV is difficult to evaluate (UNODC 2021, p. 23; UNODC 2020, p. 12; Baier et al. 2022). Nevertheless, various studies indicate a surge in domestic violence during the pandemic, especially during phases of confinement. A study commissioned by UN Women concludes that one in four women have experienced “increase household conflicts” during COVID-19 and “feel more unsafe in their home” (UN Women 2021a, p. 5). Research indicates that, in many countries, calls to helplines for domestic violence have soared during periods of confinement (UNODC 2020, p. 9; WHO Europe 2021, pp. 2 and 15; Leslie/Wilson 2020). Although lockdowns have often led to a decrease in police complaints, especially about sexual violence, this has been explained by the fact that COVID-19 mitigation measures exacerbated barriers to reporting (UNODC 2021, p. 21; UNODC 2020, p. 2). More generally, pandemic mitigation measures had an enormous impact on the accessibility and availability of mental health support, health services, and domestic abuse support, especially during lockdowns (UNODC 2020, p. 6). This was due to the closure or limitation of some services, for example general health services, as well as to other factors impeding victims to seek support, such as restricted mobility or economic insecurity (UNODC 2020, p. 6).

The difficulty to measure the concrete impact of the pandemic and its mitigation measures on IPV can be partially attributed to the general lack of comparable administrative data on gender-based and domestic violence, even outside the pandemic (UN Special Rapporteur 2020, paras 17 and 78).^17^ Limitations of crime statistics, for example, stem from varying recording measures and practices, the fact that IPV often falls under various non-specific criminal offenses (such as common assault, aggression, and coercion), and, perhaps most importantly, the fact that IPV remains notoriously under-reported (UNODC 2020, p. 10; FRA 2014, pp. 13–16 and 61). For example, an EU-wide survey in 2014 showed that only in 20% of all situations of domestic violence the “most serious incident” had been reported to the police (FRA 2014, p. 61). Under-reporting also occurs in population surveys, especially in countries where the stigma around domestic and sexual violence remains high (FRA 2014, pp. 61–64). While extremely complex at the best of times, data collection and interpretation have been hindered during COVID-19 (UNODC 2020, p. 2). Among others, comparison of available data is hindered by differing and rapidly changing state responses as well as different reference periods (WHO Europe 2021, p. 7).

Beyond COVID-19, studies have shown that situations of crisis tend to increase IPV. The phenomenon has been observed in relation to different crises: other epidemics, such as the  Ebola outbreak, but also natural disasters such as floods and earthquakes (EIGE 2021, p. 2; Mittal/Singh 2020, pp. 2–3; Peterman et al. 2020, pp. 10–11), armed conflict, or forced migration (de Oliveira Araujo et al. 2019, p. 10; Peterman et al. 2020, p. 10). Based on an extensive literature review, one study identifies nine main pathways or reasons why pandemics tend to increase gender-based violence: economic insecurity and stress; quarantine and social isolation; disaster and conflict-related unrest and instability; destabilization of gender norms and roles; inability to temporarily escape abusive partners; virus-specific sources of violence (such as withholding of sanitary equipment); reduced access to health services and first responders; exposure to violence and coercion due to efforts to respond to the pandemic or crisis; and violence perpetrated against health care workers, including domestic violence (Peterman et al. 2020, pp. 5 and 14; see also WHO Europe 2021, p. 2).

All these elements tend to exacerbate vulnerabilities in relation to gender-based violence. Indeed, whereas proponents of vulnerability theory have taken great care to emphasize the common, shared vulnerability of all human (and, possibly, all living) beings, they have also stressed that vulnerabilities are unequally distributed.^18^ Within human rights law, vulnerability is best understood as the interaction of two factors: risk (to have one’s rights violated) and resilience — that is, a person’s means allowing them to anticipate, withstand, cope with, and generally react to such violations (Zimmermann 2022, pp. 92–98, with further references).^19^ Resilience, in turn, encompasses the sum of material, human, social, environmental, and existential resources at a person’s disposal, which are influenced by historically entrenched patterns of discrimination, social inequalities, and structures of power (Fineman 2008, pp. 13–14; Fineman 2017, p. 146; Becerra 2012, p. 10; Zimmermann 2022, pp. 94–95). Resilience should, thus, be understood in a structural and societal sense to avoid burdening victims with misled notions of individual responsibility, which can lead to victim-blaming (Cole 2016, pp. 137–152). Thus, individual and structural factors of vulnerability interact and mutually shape each other. In relation to IPV, vulnerabilities are the result of the interaction between context-specific risk factors (such as alcohol consumption or stress) as well as more structural elements (such as poverty or gender) and the presence or absence of resilience-building institutions or mechanisms. The concepts of risk, resilience, and vulnerability also allow us to analyze how different situations, such as crises, impact both risk of victimization and the available resources or resilience-building mechanisms, such as the accessibility of help services. The nine pathways identified by Peterman et al. also underline the long-term consequences of a crisis on IPV: the socio-economic impact of the COVID-19 crisis will be felt long after lockdowns and other measures of social isolation are lifted and is thus likely to influence the prevalence of IPV in the longer term (Peterman et al. 2020, p. 8).^20^

## Crisis Framing: Exposing Vulnerabilities

While the COVID-19 pandemic has thus exacerbated vulnerabilities in relation to IPV, another phenomenon has taken place during the same period. Early in the pandemic, international institutions and other actors managed, through considerable agenda-setting efforts, to shed light on domestic violence and bring it to the forefront of public debate (UN Special Rapporteur 2020). Indeed, soon after the start of the COVID-19 pandemic, when countries around the world started introducing measures of social distancing and lockdowns to limit the spread of the virus, international institutions, and human rights bodies warned against the potential impact of such measures on IPV and on domestic violence in general (see, e.g., UN 2020, pp. 17–18; UN Women 2020; UN Special Rapporteur 2020, paras 10-24). UN agencies and human rights bodies have specifically called for a gender-sensitive implementation of measures responding to the COVID-19 crisis and the adoption of “measures to address a “horrifying surge in domestic violence cases affecting women and girls” (UN Special Rapporteur 2020, para. 10). They have also encouraged data collection monitoring the impact of the crisis on IPV against women (UN Special Rapporteur 2020, paras 76-80 and 91).

In these sustained policy-making efforts to expose IPV, crisis rhetoric has played a considerable part. In spring 2020, after the first weeks of the COVID-19 pandemic, UN agencies, human rights bodies, and NGOs started drawing attention to the risk that mitigation measures posed to domestic violence, calling the phenomenon a “shadow pandemic” (UN Special Rapporteur 2020, paras 19 and 38; UN Women 2021a). Somewhat paradoxically, the term “shadow pandemic” was a rhetorical part of the agenda-setting effort to bring IPV into the limelight (Evans et al. 2020). I argue that this was not accidental: crisis framing was pivotal in making the matter a policy priority. Indeed, the expression “shadow pandemic” forcefully links IPV to a concept that conveys a public health matter of great concern, a pandemic. The public debate has also seen more explicit crisis framing: domestic violence against women and girls has been termed a “hidden crisis” (End Violence against Children 2020) or, more generally, a “global crisis” (OECD 2022; UN Women 2021b). Thus, whereas the crisis has exacerbated vulnerabilities, crisis language has contributed to exposing them and calling for state action. This is novel only to a certain extent: indeed, crisis language has been used for some time in relation to IPV and other forms of gender-based violence.

Even before COVID-19, international organizations have pushed a crisis narrative to establish the fight against violence against women as a priority, such as when the WHO called for “worldwide action […] to address [the] hidden crisis of violence against women and girls” (e.g., WHO 2014). Much earlier, the women’s rights movements of the 1970s relied on crisis framing to push for IPV and other forms of violence against women to be recognized as human rights violations. The creation of Rape *Crisis* Centres, which still operate today, is anecdotal evidence of a larger effort to use crisis framing for feminist agenda-setting (Abrams 2018, pp. 752–757; Otto 2014, p. 89).

This is due to the discursive force of the term “crisis” (Authers and Charlesworth 2014, pp. 23–24), which conveys that a matter is bothof great concern and of great urgency, and calls for immediate and robust action (Authers and Charlesworth 2014, pp. 22 and 38; Abrams 2018, p. 751). Crisis represents a “vitally important or decisive stage” or a “turning point” (OED 2020; Abrams 2018, p. 765). At its largest, the term is used to capture any “phenomenon deemed outside normality” (D’Aspremont 2022, p. 72). Characterized by its urgency, crisis is “malleable and ambiguous”, and derives its discursive force at least partially from its lack of definition (Authers and Charlesworth 2014, p. 24). In any case, it evokes exceptionality and favors what has been called “emergency time”: a disproportionate focus on the immediate present, dealing with each crisis “on the point of eruption” and then moving on to the next, seemingly more pressing issue (Hansel 2019, p. 387).

International law has been described as a “discourse for crisis, about crisis, and in crisis” (D’Aspremont 2022, p. 71). Contemporary human rights law as such can be said to be founded on a “crisis model” (Hansel 2019, p. 381). For example, the Universal Declaration of Human Rights (UDHR) is explicitly framed as a response to one or several past crises — namely the “disregard and contempt for human rights [resulting in] barbarous acts which have outraged the conscience of mankind” (UDHR: Preamble), a formulation that refers, though not explicitly, to the atrocities of the Second War and the Holocaust (Authers and Charlesworth 2014, pp. 25–27; Hansel 2019, p. 381).^21^ A sense of crisis was also present throughout the making of the UDHR, which was “composed under the sign of emergency” (Slaughter 2007, p. 4; Authers and Charlesworth 2014, p. 20).^22^ Crisis, in such cases, “acts as a catalyst” for normative change (Authers and Charlesworth 2014, p. 28). Some authors have analyzed the same phenomenon through a vulnerability lens, concluding that “the realization of our own vulnerability is a crucial factor in the development of human rights” (Andorno 2016, p. 264; see also Grear 2010, pp. 137–167).

However, human rights law’s relationship with crisis is a fraught one. Most notably, the existence of a crisis is frequently invoked to justify limitations on human rights (Authers and Charlesworth 2014, pp. 28–30). Whereas formal derogations from human rights treaties are confined to some types of emergencies and strictly regulated, crisis discourse has led to less formal, and thus less strictly controlled, limitations.^23^ Moreover, crisis narrative is, by definition, partial and constructed and can also deflect from certain causes and issues. This aspect is particularly relevant in relation to IPV. Indeed, historically, women’s rights have been marginalized by international law’s “tendency to prioritize crisis” (Hansel 2019; Otto 2014). This is partly because a focus on crisis tends to favor civil and political rights over the economic, social, and cultural rights that would be necessary to bring about structural change and substantial equality but are seen as too commonplace to qualify for immediate redress and as deferrable (Hansel 2019, pp. 383–384; Authers and Charlesworth 2014, pp. 30–37). It is also because the systemic violence and structural discrimination that women experience on a daily basis are considered to be “part of the status quo” or quotidian matters (Charlesworth 2002, p. 389). Not being considered as crises, they tend to be side-lined and forgotten (Hansel 2019, pp. 383–385; Charlesworth 2002, pp. 389–391). To reverse this perception, defendants of women’s rights have resorted to crisis framing themselves. As Otto (2014, p. 89) forcefully explains, “a great deal of feminist legal scholarship and activism has been concerned with recasting everyday sexual violence as a crisis that must be addressed as a priority. In this way, adopting the language of crisis can be a strategy of desperation aimed at drawing attention to the everyday brutalities suffered by far too many women, hoping to propel them onto the official maps of international law and politics”. While this endeavor has been successful to a certain extent, it is not without its drawbacks, as I will show below.

## Vulnerability Rhetoric and Positive Obligations

In the following, I will demonstrate how vulnerability rhetoric has shaped positive human rights obligations in relation to IPV. I will focus on the ECtHR case law, which illustrates how vulnerability reasoning can play a role akin to crisis discourse. Before doing so, a few preliminary remarks on the European human rights framework in relation to IPV are in order. The European framework is marked by the interplay of two instruments: the Istanbul Convention, arguably the most far-reaching binding instrument on gender-based violence, and the European Convention on Human Rights (ECHR) as interpreted by the ECtHR. The ECtHR has developed, since 2007, a consistent strand of case law dealing with IPV, deducing detailed positive obligations from the right to life, the prohibition of torture and ill-treatment, and the right to private life, sometimes in link with the prohibition of discrimination (among others, see Opuz v. Turkey; Volodina v. Russia; Kurt v. Austria [GC]). Importantly, case law shows a certain trend recognizing not “only” violations of the right to private life, but also violations of the prohibition of torture and ill-treatment, thus responding to a long-standing scholarly demand (McQuigg 2011, pp. 47–48; Sjöholm 2018, pp. 426, 435; Zimmermann 2022, pp. 485–491) . The Istanbul Convention has been inspired by ECtHR case law (Council of Europe 2011, para. 29). However, its obligations are more specific and far-reaching, partly due to its general scope: whereas the ECtHR deals with individual cases, the Istanbul Convention sets out policy goals and preventive obligations. The Istanbul Convention has its own monitoring mechanism, but the ECtHR has continued to draw upon the Istanbul Convention to further develop its case law on positive obligations to prevent and combat domestic violence (Zimmermann 2022, p. 497, with further references).

Throughout ECtHR case law, vulnerability has played a role akin to crisis discourse. Indeed, just like crisis framing, vulnerability reasoning is a forceful rhetoric arguing that an issue demands priority action (Mustaniemi-Laakso and Heikkilä in this issue). The parallels run deep: indeed, authors have attributed the popularity of vulnerability to the way it captures the “Zeitgeist of modernity” with “today’s narratives on risk, crisis and terrorism” and “the idea of risk society [focusing] our attention on ‘an overwhelming feeling of uncertainty’ and the continuous exposure to future risks” (Misztal 2011, pp. 48–49). Vulnerability, then, is a forceful call for action, drawing attention to a situation where a strong state response is warranted (see the concept of a “responsive state” coined by Fineman 2010, pp. 259–262; Fineman 2017, pp. 146–149).

These aspects of vulnerability reasoning feature prominently in ECtHR case law. Indeed, the ECtHR has used vulnerability language both to underline the importance of an issue and to call for state action in the form of enhanced positive obligations (Besson 2014, pp. 65–66; Peroni and Timmer 2013; Heikkilä and Mustaniemi-Laakso 2020, pp. 791–794; Zimmermann 2015). Since the very first case concluding to a Convention violation because of a situation of IPV, the ECtHR has consistently emphasized the “particular vulnerability of the victims of domestic violence and the need for active State involvement in their protection” (Bevacqua and S. v. Bulgaria, para. 65; see also, similarly, Opuz v. Turkey, para. 160, Volodina v. Russia, para. 72).

In particular, the Court has relied on vulnerability reasoning to firmly refute any considerations according to which IPV would be a “private” affair, highlighting the state’s role in actively protect against such violence (Bevacqua and S. v. Bulgaria, para. 65). In later cases, the Court has used vulnerability to develop and concretize the state’s positive obligations necessary to ensure the effective protection of the human rights guaranteed by the ECHR (e.g., Bălşan v. Romania, para. 57; Volodina v. Russia, para. 77). Over the years, the Court has fleshed out three categories of positive obligations: to adopt an adequate legislative framework, to carry out a prompt and effective investigation, and to take operational measures for protection and prevention (Zimmermann 2022, p. 484; see e.g. Bălşan v. Romania, para. 57; Volodina v. Russia, para. 77).^24^ While these positive obligations also apply to other instances of private violence, they are particularly significant and far-reaching concerning the protection of vulnerable individuals (Zimmermann 2022, p. 492; see e.g. Opuz v. Turkey, para. 159).^25^

The first positive obligation can be summarized as follows: the legal framework must allow to effectively prevent, prosecute and punish domestic violence. The adoption of a framework punishing acts of domestic violence not only serves a repressive aim, but also has a preventive function, because of the deterrent effect expected from such legislation.^26^ Obligations relating to the legal framework encompass first and foremost the obligation to criminalize acts of domestic violence, even if they do not result in physical violence (Ž.B. v. Croatia, para. 50; Volodina v. Russia, para. 78). The Court has justified the need for criminal law remedies, which are alone considered sufficiently deterrent (Ashworth 2013, pp. 201 and 209–210), by the vulnerability of victims of domestic violence, calling for active state involvement (Volodina v. Russia, para. 78), and the “increasingly high standard[s]” of human rights protection, requiring “greater firmness in assessing breaches” (A v. Croatia, para. 67). The Court has also held that the criminal legal framework must allow for the ex officio prosecution of domestic violence (Volodina v. Russia, para. 82).

Whereas ex officio prosecutions protect the victim against pressure from violent spouses or authorities to withdraw their complaint, they are paternalistic to a certain extent (Zimmermann 2022, p. 505; Goodmark 2018, pp. 14, 20, and 115). The difficult balancing act between respecting the victim’s autonomy and guaranteeing the effectiveness of conventional rights also cautions against focusing on criminal law remedies (Zimmermann 2022, pp. 505–506; Goodmark 2018, pp. 17–23 and 110–111). Indeed, in the case law of the Court, criminal law lies at the center of legislative obligations, which is unsatisfactory (Lavrysen 2014, p. 125; Tulkens 2011, pp. 577–595). While the Court’s findings regarding legislative obligations are necessarily limited by its case-by-case, subsidiary role,^27^ it would be desirable for the Court to give more concrete expression to legislative obligations that do not fall within the scope of criminal law. A close reading of the existing case law indicates a few requirements: the legal framework must more generally provide for preventive measures, such as perimeter or contact bans, and, more generally, form the basis for a rapid response by the authorities (A v. Croatia; Buturugă v. Romania; Volodina v. Russia). The Court could draw further inspiration from the Istanbul Convention’s framework and elaborate on obligations of sensibilization and prevention and the creation of effective help services, such as telephonic helplines and sufficient and accessible shelters (de Vido 2020, pp. 57–74; Peroni and Timmer 2013, pp. 62–63; Volodina v. Russia, op. diss. Pinto de Albuquerque, paras 16–18 and 20). The Court could, for example, reverse the burden of proof of allegations of discrimination in the absence of satisfactory preventive mechanisms (for this suggestion, see Zimmermann 2022, pp. 566–567).

The second positive obligation is to carry out a prompt and effective investigation when the state is confronted with indications of domestic violence. This obligation has led to various findings of Convention violations in the case law (M. and M. v. Croatia, para. 136; Volodina v. Russia, para. 77). One of the biggest problems is the passivity of the authorities, who are slow to open an investigation or more generally to act, do not take the necessary steps to properly establish the facts, or lack due diligence in managing the proceedings. Sometimes this passivity is based on an explicitly condescending attitude on the part of the authorities. In several cases, the Court has found that the authorities’ failure to act violated the prohibition of discrimination enshrined in Article 14 ECHR (see, e.g., Eremia v. Moldova; T.M. and C.M. v. Moldova; Bălșan v. Romania; Mudric v. Moldova; Volodina v. Russia; Talpis v. Italy).

The third positive obligation flowing from ECtHR is the duty to take preventive and protective measures. This obligation arises when the authorities had or should have had knowledge of a sufficiently serious risk of harm to the life or physical or psychological integrity of a person (Osman v. The United Kingdom, paras 115–116). The Court’s standard is that of a “real and immediate risk”, which it has so far considered to be applicable even in cases of IPV or domestic violence, despite voices arguing for a lower standard of a “real and present risk” (Volodina v. Russia, paras 86–87; Kurt v. Austria [GC], para. 164; Valiulienė v. Lithuania, op. diss. Pinto de Albuquerque; de Vido 2020, pp. 64–65). However, the Court has emphasized, in a series of cases, that the authorities must duly consider the vulnerability of victims of domestic violence and assess the existing risk in light of the specific nature of domestic violence (Talpis v. Italy, para. 122; Volodina v. Russia, para. 86; Kurt v. Austria [GC], paras 175–176). Referring to the Istanbul Convention, the Court has also highlighted that the authorities must conduct a detailed risk assessment (Kurt v. Austria [GC], paras 167–176). Various instruments exist to assist the authorities in this endeavor, such as the Spousal Assault Risk Assessment or the Multi-Agency Risk Assessment Conference. Among the risk factors to consider are parameters such as an increase in the frequency or intensity of violence, the possession of weapons, drug use or alcohol abuse, or a recent separation or denunciation, as all these factors have been known to increase a victim’s risk of further victimization (Zimmermann 2022, p. 464). In the context of domestic violence, a repetition of violence is very likely to occur. Indeed, long-standing psychosocial and criminological research shows that even in the absence of explicit threats, victims of domestic violence are at increased risk of experiencing further violence, because violence often follows a “cycle of violence”, worsening over time, and leading to — sometimes sudden — escalation (Grdinic 1999, p. 239; Meyersfeld 2012, p. 123). This means that an analysis that is sensible to the specific nature of IPV will establish the existence of a risk — whatever the formal threshold — sufficient to trigger further action (but: cf. Kurt v. Austria [GC], paras 203–211; for further information, see Zimmermann 2022, pp. 523–541). This, then, triggers the related positive obligation to take “all reasonable measures” to avoid the risk from realizing. This obligation is much less far-reaching than it seems at first glance: indeed, in many cases, it simply signifies that the authorities will have to effectively apply in practice the existing legal framework.

It is difficult to deduce specific guidelines from the Court’s case law regarding potentially enhanced positive obligations during the COVID-19 pandemic. This is even more so because the ECtHR’s role and scope is geared towards individual, rather than structural, remedies. Despite that, the case law gives some general guidance. First, following the Court’s logic, exacerbated vulnerability (due to COVID-19) should give rise to enhanced positive obligations. Second, the obligation to conduct a detailed risk assessment considering all relevant risk factors is particularly relevant during the pandemic and involves considering pandemic-specific risk factors. Third, even in times of pandemic, states are under the obligation to ensure the effectiveness of Convention rights, which also implies the effective availability of prevention and protection mechanisms. While the ECHR allows for “derogations in times of emergency”, these are strictly limited. An emergency needs to be such as to “threate[n] the life of the nation”, the measures need to be “strictly required by the exigencies of the situation”, consistent with other obligations under international human rights law and cannot concern the Convention’s non-derogable rights (Article 15). It has also been argued that derogations should make specific provisions for the protection of vulnerable persons, lest they be disproportionate (Lebret 2020, p. 9). Even if the pandemic would meet the restrictive criteria set out by Article 15 ECHR, which is doubtful, they would only concern a minority of cases on IPV — namely those filed under the right to private and family life (Article 8). Indeed, many IPV concern the right to life (Article 2) or the prohibition of torture and other ill-treatment (Article 3), which are both non-derogable rights (Article 15(2)). Somewhat more boldly, one could wonder whether there might be a positive obligation to take preventive measures to mitigate the obstacles that COVID-19 imposed on prevention and protection mechanisms, for example, by increased preventive action (see also Lebret 2020, pp. 9–12).

## Vulnerability Seen Through a Crisis lens

Considering vulnerability’s important role in the ECtHR’s case law on IPV, it is perhaps surprising that the Court gives little guidance as to how this vulnerability is to be understood. Indeed, with a few notable exceptions that will be discussed below, the Court mostly states that victims of domestic violence are vulnerable, sometimes referring to past violence and indicating that this vulnerability is due to victimization (Rumor v. Italy, para. 60; M.G. v. Turkey, para. 105). This approach has been described as a form of “ex-post vulnerability” (Sjöholm 2018, p. 433; Timmer 2013, p. 175). In a few cases, the Court also mentions the victim’s age or physical fragility as a source of vulnerability (Mudric v. Moldova, para. 51). In a more prospective manner, some cases explicitly link past victimization to a person’s individualized risk of (re-) victimization (Rumor v. Italy, para. 60).

The ECtHR mostly tends to simply highlight the vulnerability of victims of IPV, in an ex-post approach that conveys a simplified and somewhat static image of vulnerability akin to a “label” (Luna 2009). The Court’s emphasis lies not so much on why the applicants in any given case are vulnerable, but on what their vulnerability does for the legal reasoning: establishing the state’s responsibility and need for action via the doctrine of positive obligations. Literature on crisis discourse explains why vulnerability language is powerful: emphasizing someone’s vulnerability is akin to evoking a “moment of crisis” and suggests a sense of urgency and exceptionality, calling for action. However, labeling some groups and persons as vulnerable to argue for their protection has significant drawbacks. The risks of paternalism, essentialism, othering, and stereotyping have been extensively discussed in topical scholarship (among many others, see Mackenzie et al. 2014, pp. 1–26; Morondo Taramundi 2016, pp. 206–210; Peroni and Timmer 2013, pp. 1071–1074; Zimmermann 2022, pp. 83–92, with further references). In this article, I argue that apprehending vulnerability reasoning as a form of crisis discourse can improve our understanding not only of the appeal of vulnerability language, but also of some of its most salient risks.

To illustrate the risks of vulnerability seen through a crisis lens, it is worth considering the criticisms raised against crisis framing. Otto forcefully demonstrates that while some feminist attempts to reclaim crisis framing have been successful, the solutions favored by a crisis narrative tend to neglect core feminist values (Otto 2014, pp. 89–92). The crisis model favors a narrowly defined and straightforward narrative, which often neglects “contextualization in history, culture, past feminist campaigns, or present specific realities”, thereby reinforcing potentially stereotyping narratives of “us versus them” such as men as perpetrators, women as victims, or otherwise essentializing conceptions (Otto 2014). Vulnerability reasoning similarly creates narrow, stereotypical, and essentializing views of “the vulnerable” and “the others”. The focus on vulnerability due to past experiences or ex-post vulnerability, notably, risks stereotyping victims of IPV as being inevitably and irremediably vulnerable. Moreover, the crisis model calls for immediate, straight forward, short-term, and narrowly focused responses (Authers and Charlesworth 2014; Otto 2014). Crisis narrative tends to champion medical and “law and order” responses over social and structural reforms, favoring criminal law remedies against domestic violence instead of addressing the root causes and taking preventive social action (Otto 2014, p. 92; see also Abrams 2018, pp. 757–759). This may result in overly repressive and potentially paternalistic policies, which can be framed as sources of pathogenic vulnerability (Mackenzie et al. 2014, pp. 9 and 12–13). A crisis approach to vulnerability likewise favors potentially paternalistic approaches or policies generating pathogenic vulnerabilities, including overcriminalization (Zimmermann 2022, pp. 83–91). Crenshaw’s analysis of the way that “law and order” responses to domestic violence, such as increased policing, mandatory arrests and higher penalties, affect communities of color, is a poignant illustration of such pathogenic vulnerabilities elicited by a crisis response to domestic violence (Crenshaw 2012, pp. 1452–1457).

Perhaps most importantly, a crisis approach tends to over-emphasize some vulnerabilities — those framed as a crisis or emergency — while overlooking others. In this vein, vulnerability reasoning has been criticized for hierarchizing needs and rights, thus neglecting persons seen as being “not vulnerable enough”.^28^ This, then, burdens the victim with “performing” their vulnerability, failing which she loses the enhanced protection that comes with it (Kapur 2002, p. 10; Peroni 2016, pp. 50–53 and 59–61). For example, concerning a migrant woman who had experienced different forms of gender-based violence in her country of origin, Peroni pervasively shows how the ECtHR highlights the applicant’s “strength”, “assertiveness”, “resourcefulness”, and overall non-vulnerability to conclude that her deportation did not violate her human rights (Peroni 2008, pp. 359–363; also see A.A. v. Sweden).

In short, while it is tempting to read vulnerability through a crisis lens calling for protection of “the most vulnerable”, such a “humanitarian approach” (Carron et al. 2021) is directed toward short-term relief instead of working towards long-term structural change.

## Beyond Crisis-Discourse: A Nuanced Approach to Structural Vulnerabilities

However, a more nuanced approach is possible. In relation to crisis, Charlesworth and Hansel suggest replacing emergency time by an understanding of “time as repetition”, creating an “international law of the everyday”, concerned with “enduring, pervasive issues relevant to people’s lives on a daily basis — such as economic inequalities, healthcare, domestic violence, racial discrimination” (Hansel 2019, p. 393). Paradoxically, and despite the criticism voiced above, vulnerability reasoning is a useful tool for such a more nuanced analysis on the condition that vulnerability itself is understood as a relational and dynamic concept, considering a person’s personal, social, and institutional situation (Peroni 2016, p. 62; Zimmermann 2022, pp. 469–471). When vulnerability is not understood as a “given”, but as the result of interacting power structures that shape a person’s life experience, it invites us to pay closer attention to the root causes of human rights violations.^29^ Focusing on the underlying structural causes of various overlapping and interacting vulnerabilities invites us to leave behind the crisis narrative’s “emergency time”, adopt another temporality and focus on creating resilience-building institutions in the long term (Fineman 2008, 2017).

Beginnings of such an approach can be found even within the Court’s current case law on IPV, and future case law could — and should — elaborate on them. Indeed, the Court’s somewhat “label-like” approach to vulnerability is partly compensated by the fact that the Court also highlights the relevance of each applicant’s individualized, context-specific risk factors, specifically stating that authorities were under the positive obligation to conduct a holistic and circumstantial risk assessment of their own initiative (Kurt v. Austria [GC]). Various frameworks exist for such an analysis, such as the Spousal Assault Risk Assessment or the Multi-Agency Risk Assessment Conference.^30^ In this regard, the Court also underlines the necessity of a proactive and autonomous approach by the relevant authorities, which goes some way toward creating a “responsive state” (on the idea of a responsive state, see Fineman 2010, pp. 259–262; Fineman 2017, pp. 146–149).

Even though risk assessment remains essentially focused on the individual’s specific situation, a departure from crisis time and a more nuanced and far-reaching understanding of vulnerability also needs to consider the structural and systemic sources of vulnerability. Acknowledgements of such sources by the ECtHR are far and few between; nevertheless, existing case law contains a few promising starting points. In Opuz v. Turkey (para. 159), the Court mentions the applicant’s socio-economic status and, indirectly, her ethnicity. In Talpis v. Italy (paras 25–28 and 83), the Court alludes to the applicant’s Moldovan and Romanian nationality and statistics indicating an even higher prevalence of domestic violence in these countries. However, it does not consider other, more relevant structural vulnerability factors, such as the applicant’s situation as a migrant woman recently arrived in Italy, without employment or independent financial means.^31^

In this regard, both the relevant literature and human rights bodies cite migration status as a factor of structural vulnerability, particularly because of the social isolation, material and legal precariousness, and experiences of discrimination or stigmatization it often entails (Condon et al. 2011, pp. 68–70; Meyersfeld 2012, pp. 129, 179; Lehmann 2011, p. 98).^32^ All of these elements can obstruct the victim’s access to means of resilience, such as effective access to justice and are elements to which both the Court and domestic authorities should be attentive to fulfill their obligation “to take into account the situation of precariousness and particular vulnerability, moral, physical and material, in which the applicant found herself and to assess the situation accordingly, by offering her appropriate support” (Talpis v. Italy, para. 115). Importantly, vulnerability analysis calls for a nuanced approach, examining how institutional and legal barriers create and exacerbate vulnerability, to avoid essentializing and othering migrant women (Montoya and Rolandsen Agustín 2013, pp. 534–557; Peroni 2016, pp. 53–55).

Gender is another structural source of vulnerability in relation to IPV. Perhaps not surprisingly, the Court has been reluctant to recognize gender as a source of structural vulnerability, going so far as to stress that it was “unable to fully share the applicant’s view that she, as a woman, by default fell into the category of vulnerable persons” (Valiulienė v. Lithuania, para. 69). The Court is not alone in hesitating to qualify women per se as particularly vulnerable, even within the very specific context of domestic violence. Some authors fear that this qualification could reinforce gender stereotypes portraying women as “inherently” “weak” or unable to defend themselves (Kapur 2002, p. 36; Peroni 2016, p. 52; Peroni and Timmer 2016, pp. 47–48).^33^ However, I consider the recognition of gender as a source of vulnerability as a useful step towards a gender-sensitive analysis of IPV and of the state’s positive obligations. Implicitly, the Court has already acknowledged this, by recognizing the gendered nature of domestic violence (Volodina v. Russia, para. 110; Tkhelidze v. Georgia, paras 76–77) and by finding a discrimination contrary to Article 14 ECHR in several domestic violence cases (see, e.g., Opuz v. Turkey, paras 199-202; Eremia v. Moldova, paras 85–90; Bălșan v. Romania, paras 78–89). Reasoning explicitly in terms of structural vulnerability would allow the Court to elaborate on and further enhance its positive obligations in the field.

I argue that a more explicit judicial reasoning on these sources of individual and, most importantly, structural vulnerability would serve a twofold goal. First, it would allow for a more nuanced understanding of vulnerability and avoid its most salient drawbacks, notably the risks of stereotyping and essentializing. Second, with vulnerability being a powerful rhetoric, it would justify the Court’s current case law on enhanced positive obligations and allow the Court to take a few steps further. It could, for example, concretize the non-criminal aspects of the positive obligation to adopt an adequate legal framework, drawing inspiration from the Istanbul Convention. It could also elaborate on preventive structural obligations via the concept of non-discrimination. For example, a state’s failure to take structural measures against gender-based discrimination could reverse the burden of proof as to the existence of discrimination in cases of IPV (for a more detailed argumentation on this point, see Zimmermann 2022, p. 511). While the Court is necessarily limited by its role as an international judicial organ, bound by the principle of subsidiarity and obliged to leave a considerable margin of appreciation to the states as to how to concretize and implement their Convention obligations (Brems 2019, pp. 210–227; Valiulienė v. Lithuania, para. 85; see also the Preamble of the Convention as modified by Protocol no. 15), it has a crucial role in guaranteeing the effectiveness of the rights enshrined in the Convention. Part of this role is to ensure the responsiveness of states via the doctrine of positive obligations.

## Conclusion

In this article, I have examined the links between vulnerability and crisis in relation to IPV. I have started by introducing IPV as a human rights issue, affecting various human rights. I have then shown that the COVID-19 pandemic has exacerbated existing vulnerabilities and created new ones, by accentuating and multiplying risk factors while limiting resources at the victims’ disposal. I have placed this phenomenon within the wider context of pandemic literature, indicating that pandemics — just like other crises — are known to exacerbate vulnerability in relation to gender-based violence. Having examined the relationship between crisis and IPV, I then turned to crisis discourse*.* Using examples from several actors, I showed how crisis rhetoric has been mobilized to shed light on IPV and call for a more active and robust response. Crisis discourse is neither recent nor limited to the context of COVID-19: on the contrary, it is intimately linked to human rights law and has also contributed to IPV being recognized as a human rights issue. I have then drawn a parallel between crisis discourse and vulnerability reasoning, indicating that both are powerful rhetorical devices conveying a sense of emergency and are therefore used to insist upon and strengthen states’ human rights obligations, often in the form of due diligence or positive obligations. I have also examined the fraught relationship between crisis discourse and women’s rights — in this case, intimate partner violence, understood as a specific form of gender-based violence. Feminist authors have criticized the nefarious impact of international human rights law’s fascination with crisis on these topics, seen as “too quotidian” to warrant attention. They have also argued that reclaiming crisis discourse to further women’s rights is a risky endeavor, as it might favor crisis-driven responses instead of long-term structural reforms. Whereas vulnerability reasoning can have similar drawbacks, this depends on the understanding of vulnerability. If it is true that vulnerability sometimes functions as a specific version of crisis rhetoric — an evocative label indicating that a strong response is warranted — another, more holistic approach to vulnerability is possible. If we avoid seeing vulnerability through a crisis lens, focusing instead on a relational, dynamic, and, most importantly, structural understanding of vulnerability, it can be an important tool for law-making and a responsive state. While sharing the same appeals of crisis discourse, mainly its evocativeness and strong rhetorical force, it manages to avoid at least some of its pitfalls. Although not without its drawbacks, vulnerability reasoning can be a path away from emergency time, leading the way towards lasting structural change.

## Footnotes

1 Whereas the cited statement specifically concerns violence against children, other sources, news outlets in particular, have used it in relation to IPV (MSNBC 2020). Moreover, IPV and gender-based violence have been termed a “hidden crisis” long before COVID-19, see WHO (2014) Worldwide action needed to address hidden crisis of violence against women and girls, Press release, 21 November 2014.

2 For an analysis of the gendered impact of COVID-19, see Branicki (2020).

3 In the following, I use the term positive obligations to refer to all state obligations that go beyond mere abstention, including obligations of result (i.e., the obligation to criminalize) as well as due diligence obligations. For a discussion of the nuances, which would go beyond the scope of the present article, see Stoyanova (2020, pp. 95–129), Grans (2018, pp. 735–746).

4 For a telling example of this argument, see the tweet by UN Special Procedures, 23 November 2020 (“#COVID19 is overshadowing the pandemic of femicides and gender-based violence against women and girls — UN expert @DubravkaSRVAW calls for the establishment of national femicide watches or observatories around the world to prevent such killings”).

5 Coined by Mackenzie, Rogers, and Dodds, the expression “pathogenic vulnerabilities” refers, amongst others, to policies responding to certain vulnerabilities that, in the process, exacerbate or generate other vulnerabilities (Mackenzie et al. 2014, pp. 9 and 12–13).

6 On specific forms of psychological violence, for example a partner’s weight control, see Obreja (2020, pp. 1207–1221).

7 I consider the term “women” to encompass all persons who identify, entirely or partially, as women as well as all persons who are legally or socially perceived as women.

8 For a critical appraisal, indicating the need to analyze IPV from an intersectional perspective, see Obreja (2019, pp. 64–66, 73–80).

9 The recognition of the gendered nature of violence also facilitates the recognition of human rights violations flowing from secondary victimization due to administrative violence faced by survivors (see e.g. J.L. v. Italy, paras 117–125).

10 CEDAW (1992), General Recommendation No. 19 on Violence against women, A/47/38, para. 1.

11 Special Rapporteur on violence against women and girls. https://www.ohchr.org/en/special-procedures/sr-violence-against-women. Accessed 12 August 2022.

12 Office of the Hight Commissioner on Human Rights (OHCHR) Working Group on discrimination against women and girls. https://www.ohchr.org/en/special-procedures/wg-women-and-girls. Accessed 12 August 2022.

13 Special Rapporteur on violence against women and girls. Platform for strengthening cooperation between international and regional independent mechanisms on violence and discrimination against women. https://www.ohchr.org/sites/default/files/Documents/Issues/Women/SR/CooperationbetweenGlobalRegionalMechanisms.pdf. Accessed 12 August 2022.

14 CEDAW (2017), General Recommendation No. 35 on gender-based violence against women updating General Recommendation No. 19, CEDAW/C/GC/35.

15 Depending on the concrete context of IPV, a myriad of civil and political as well as socio-economic rights may be violated : the right to life, the prohibition of ill-treatment, the right to private and family life, the right to health, etc. (see also Obreja 2019, pp. 63–80, with a focus on socio-economic rights).

16 Refuting the public/private divide of civil rights and liberties as well as their focus on negative obligations has been a major theme of second-wave feminism (Hanisch 1970, p. 76; Kelly 2022).

17 Assessing the “availability, quality and comparability of administrative data” across EU member states, the European Institute for Gender Equality (EIGE) has identified the police and justice systems as sources of the most comparable data. However, limitations arise from varying recording practices and measures (incidents, reports, or crimes), and the fact that domestic violence and IPV correspond to different crimes and offenses, obscuring them in criminal statistics (EIGE 2016, p. 19-21; WHO Europe 20211, p. 7).

18 Universal vulnerability has been most forcefully theorized by Martha Albertson Fineman, arguably the leading vulnerability theorist (see, among others, Fineman 2008, 2013, 2017). On the unequal distribution of vulnerabilities, see, among others, Casalini 2018, pp. 37–42; Morondo Taramundi 2016, pp. 213–218. Note that Martha Albertson Fineman’s position on particular vulnerabilities fluctuates somewhat. In the past, she has hold both that vulnerability is universal, but its experience is particular and that vulnerability is always universal and only resilience varies (Fineman 2012, p. 1754; see also the account of a live presentation by Morondo Taramundi 2016, p. 215). More recently, she seems somewhat more open to the idea of particular, and therefore unequally distributed, vulnerabilities (see Fineman 2017, pp. 143–144; Fineman 2019, pp. 86–88).

19 The use of the concept of risk in relation to vulnerability is somewhat controversial among vulnerability theorists. Fineman, for example, is opposed to the term because she considers that it relates to a narrowly construed imagery of prejudice (Fineman 2012b, pp. 105–106). Despite that, I consider the term to be useful and, indeed, indispensable for a human rights vulnerability analysis (see also Zimmermann 2022, pp. 94–95).

20 Some quantitative studies indicate the importance of socio-economic factors. For example, a representative study in the canton of Zurich (Switzerland) has shown that during the first lockdown, IPV remained stable in households that were not affected by partial unemployment measures but increased in households affected by COVID-19-related redundancy (Baier 2020). This indicates that it is not only the most visible aspects of a crisis (e.g., lockdown measures) that contribute the most to exacerbating vulnerabilities.

21 An earlier draft contained explicit references to both the First and the Second World War (Commission on Human Rights Drafting Committee, Report of the Drafting Committee to the Commission on Human Rights, UN Doc. E/CN.4/21, 1 July 1947, para. 49; Authers and Charlesworth 2014, p. 25).

22 Note, however, that this narrative has also been criticized, especially because it deflects from more long-term developments, ignoring human rights’ “broader historical genealogy” (Burgers 1992, p. 567; Authers and Charlesworth 2014, p. 28).

23 One example of this would be the counter-terrorism measures in the aftermath of 9/11, which have eroded several well-established human rights, including the right to liberty and a fair trial, the prohibition of torture, or the prohibition of discrimination (see also Authers and Charlesworth 2014, p. 30).

24 These obligations cover many but by far not all aspects of a due diligence framework and are generally less far-reaching. This is due to the nature and role of ECtHR case law, which is geared towards providing remedies in cases of individual violations rather than determining programmatic and structural measures. Within European human rights law, these obligations are complemented by the more detailed framework of the Istanbul Convention, which covers a range of obligations relating to prevention, protection, prosecution and coordinated policies. For a similar due diligence framework pertaining to soft law, see Aziz and Moussa (2016).

25 For an in-depth analysis of the ECtHR case law on vulnerability, see Heri (2021) and Zimmermann (2022).

26 See however, for a critical appraisal of the relationship between human rights and criminal law, Tulkens (2011, pp. 577–595).

27 However, clearly defined legislative obligations can be seen as fleshing out the principle of subsidiarity, which not only accentuates the states’ primary responsibility but also comprehends effective guidance by the ECtHR (Lavrysen 2014, pp. 127–129; Zimmermann 2022, pp. 512–513).

28 Such a “race to the bottom” can be observed in the field of migration and asylum law: with policies increasingly focusing on the needs of refugees who are deemed to be especially vulnerable, the needs — and, indeed, vulnerabilities — of those who fall outside pre-defined categories of vulnerability, most notably young, able-bodied male migrants, are often neglected or inadequately addressed (Costello and Hancox 2016, pp. 383–384; Zimmermann 2022, p. 91).

29 For a critical analysis of institutional as well as scholarly engagement with the root causes of human rights violations, a debate that lies outside this article’s scope, see Marks (2011, pp. 57–78).

30 In Kurt v. Austria, the Group of Experts on Action against Violence against Women and Domestic Violence (GREVIO) submitted a third-party intervention in which it highlighted the existence and usefulness of these risk assessment frameworks, an aspect that was taken up by the Court. This further highlights the interactions between the ECHR and the Istanbul Convention in the European framework on IPV.

31 On migration as a source of vulnerability, see Baumgärtel (2020, pp. 12–29).

32 In a similar vein, migrants are described as “persons made vulnerable by particular circumstances” (Article 12 para. 3 of the Istanbul Convention; see also Council of Europe 2011, paras 87 and 120.

33 There is a more general fear that vulnerability might be associated to weakness, thereby stereotyping the persons qualified as such (Cole 2015, p. 147). To counterbalance this risk, vulnerability theorists have emphasized the agency of the vulnerable subject, rejecting in the process the association between vulnerability and victimhood (Cole 2016, pp. 262–265 and 269–271). In a similar endeavor, various feminist authors reject the term « victim » altogether in favor of the term « survivor ». However, the latter can also pressurize victims into leaving behind the past, thus leaving little space for ongoing trauma (Cole 2016, p. 262; Schneider 2000, p. 75).
